# (*E*)-*N*-Benzyl­ideneadamantan-1-amine

**DOI:** 10.1107/S1600536812011415

**Published:** 2012-03-21

**Authors:** Xu-Dong Jin, Xue-Yue Yin, Hai-Bo Wang, Xiao-Hong Chang, Yue-Hong Jin

**Affiliations:** a College of Chemistry, Liaoning University, Shenyang 110036, People’s Republic of China; b Liaoning Provincial Institute of Measurement, Shenyang 110004, People’s Republic of China

## Abstract

In the title compound, C_17_H_21_N, the dihedral angle between the benzene ring and the imine group (—N=) is 5.1 (4)°. In the adamantane group, the C—C—C bond angles range from 107.88 (19) to 111.33 (17)°. Only weak van der Waals inter­actions contribute to the contribute to the packing of the molecules in the crystal..

## Related literature

For the synthesis and crystal structure of *N*-(4-chloro­benzyl­idene)-1-adamantyl­amine, see: Zhao & Feng (2005 ▶). For the synthesis and application of metal complexes with adamantane-ring-containing Schiff bases, see: Jin *et al.* (2011 ▶).
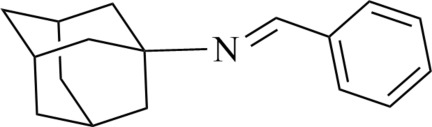


## Experimental

### 

#### Crystal data

C_17_H_21_N
*M* *_r_* = 239.35
Orthorhombic, 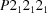

*a* = 6.480 (2) Å
*b* = 7.141 (2) Å
*c* = 29.674 (11) Å
*V* = 1373.1 (8) Å^3^
*Z* = 4
Mo *K*α radiation
μ = 0.07 mm^−1^
*T* = 296 K
0.33 × 0.29 × 0.22 mm

#### Data collection

Bruker SMART CCD area-detector diffractometer
Absorption correction: multi-scan (*SADABS*; Sheldrick, 1996 ▶) *T*_min_ = 0.978, *T*_max_ = 0.986
4971 measured reflections
2726 independent reflections
1981 reflections with *I* > 2σ(*I*)
*R*_int_ = 0.025

#### Refinement

*R*[*F* ^2^ > 2σ(*F* ^2^)] = 0.050
*wR*(*F* ^2^) = 0.140
*S* = 0.99
2726 reflections
163 parameters
H-atom parameters constrained
Δρ_max_ = 0.18 e Å^−3^
Δρ_min_ = −0.17 e Å^−3^

### 

Data collection: *SMART* (Bruker, 2004 ▶); cell refinement: *SAINT* (Bruker, 2004 ▶); data reduction: *SAINT*; program(s) used to solve structure: *SHELXS97* (Sheldrick, 2008 ▶); program(s) used to refine structure: *SHELXL97* (Sheldrick, 2008 ▶); molecular graphics: *XP* in *SHELXTL* (Sheldrick, 2008 ▶); software used to prepare material for publication: *SHELXTL*.

## Supplementary Material

Crystal structure: contains datablock(s) I, global. DOI: 10.1107/S1600536812011415/gk2442sup1.cif

Structure factors: contains datablock(s) I. DOI: 10.1107/S1600536812011415/gk2442Isup2.hkl

Supplementary material file. DOI: 10.1107/S1600536812011415/gk2442Isup3.cml

Additional supplementary materials:  crystallographic information; 3D view; checkCIF report

## Figures and Tables

**Fig. 1.**
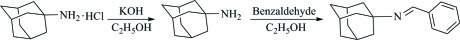
Synthetic route to the title compound.

**Fig. 2.**
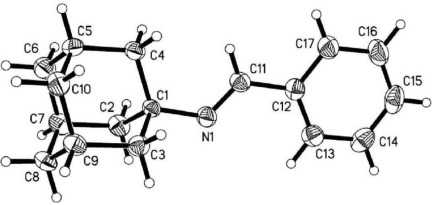
The molecular structure of the title compound with displacement ellipsoids drawn at the 30% probability level.

## supplementary crystallographic information

### Comment

The field of Schiff bases and their complexes was rapidly developing mainly owing to facile synthesis and technological applications in many areas, such as biological activity (Jin *et al.*, 2011). As an extension of our work on the structural characterization of Schiff base compounds containing an adamantane group, we synthesized the title compound (Fig.2). In the crystal of title compound (see Fig.2), the carbon atoms from the adamantane cage are *sp*^3^ hybridized with C—C—C angles ranging from 107.88 (19)° to 111.33 (17)°. The N1=C11 double bond length of 1.240 (3) Å and the C11—C12 single bond length [1.480 (3) Å] are roughly close to another set of conjugation system with C=N group [1.266 (2) Å] and C_aryl_—C(=C) bond length [1.474 (2) Å] (Zhao & Feng, 2005), respectively.

### Experimental

Amantadine hydrochloride (0.56 g, 3.0 mmol) and KOH (0.17 g, 3.0 mmol) in 50 ml anhydrous alcohol were stirred for 2 h. The produce white precipitate was filtered out and the transparent liquid was added dropwise to benzaldehyde (0.32 g, 3.0 mmol) in 30 ml anhydrous alcohol under constant stirring. The resulting solution was refluxed for *ca*. 4 h, concentrated to about 20 ml through reduced pressure distillation and then stood at room temperature. Colorless plate-shaped crystals suitable for X-ray analysis were obtained after one week by the slow solvent evaporation method.

### Refinement

The C-bound H atoms were positioned geometrically with C—H = 0.93–0.98 Å, and allowed to ride on their parent atoms with *U*_iso_(H) = 1.2 *U*_eq_(C).

### Crystal data

**Table:**

C_17_H_21_N	*D*_x_ = 1.158 Mg m^−^^3^
*M**_r_* = 239.35	Melting point: 320.5 K
Orthorhombic, *P*2_1_2_1_2_1_	Mo *K*α radiation, λ = 0.71073 Å
Hall symbol: P 2ac 2ab	Cell parameters from 5300 reflections
*a* = 6.480 (2) Å	θ = 2.8–26.4°
*b* = 7.141 (2) Å	µ = 0.07 mm^−^^1^
*c* = 29.674 (11) Å	*T* = 296 K
*V* = 1373.1 (8) Å^3^	Plate-shaped, colourless
*Z* = 4	0.33 × 0.29 × 0.22 mm
*F*(000) = 520	

### Data collection

**Table:**

Bruker SMART CCD area-detector diffractometer	2726 independent reflections
Radiation source: fine-focus sealed tube	1981 reflections with *I* > 2σ(*I*)
Graphite monochromator	*R*_int_ = 0.025
ω scans	θ_max_ = 26.4°, θ_min_ = 2.8°
Absorption correction: multi-scan (*SADABS*; Sheldrick, 1996)	*h* = −5→8
*T*_min_ = 0.978, *T*_max_ = 0.986	*k* = −8→6
4971 measured reflections	*l* = −30→37

### Refinement

**Table:**

Refinement on *F*^2^	Secondary atom site location: difference Fourier map
Least-squares matrix: full	Hydrogen site location: inferred from neighbouring sites
*R*[*F*^2^ > 2σ(*F*^2^)] = 0.050	H-atom parameters constrained
*wR*(*F*^2^) = 0.140	*w* = 1/[σ^2^(*F*_o_^2^) + (0.0708*P*)^2^ + 0.1812*P*] where *P* = (*F*_o_^2^ + 2*F*_c_^2^)/3
*S* = 0.99	(Δ/σ)_max_ < 0.001
2726 reflections	Δρ_max_ = 0.18 e Å^−^^3^
163 parameters	Δρ_min_ = −0.17 e Å^−^^3^
0 restraints	Absolute structure: Flack, H. D. (1983). *Acta Cryst.* A39, 876–881, 1097 Friedel pairs
Primary atom site location: structure-invariant direct methods	Flack parameter: −3 (5)

### Special details

**Table:**

Geometry. All e.s.d.'s (except the e.s.d. in the dihedral angle between two l.s. planes) are estimated using the full covariance matrix. The cell e.s.d.'s are taken into account individually in the estimation of e.s.d.'s in distances, angles and torsion angles; correlations between e.s.d.'s in cell parameters are only used when they are defined by crystal symmetry. An approximate (isotropic) treatment of cell e.s.d.'s is used for estimating e.s.d.'s involving l.s. planes.
Refinement. Refinement of *F*^2^ against ALL reflections. The weighted *R*-factor *wR* and goodness of fit *S* are based on *F*^2^, conventional *R*-factors *R* are based on *F*, with *F* set to zero for negative *F*^2^. The threshold expression of *F*^2^ > σ(*F*^2^) is used only for calculating *R*-factors(gt) *etc*. and is not relevant to the choice of reflections for refinement. *R*-factors based on *F*^2^ are statistically about twice as large as those based on *F*, and *R*- factors based on ALL data will be even larger.

### Fractional atomic coordinates and isotropic or equivalent isotropic displacement parameters (Å^2^)

**Table:**

	*x*	*y*	*z*	*U*_iso_*/*U*_eq_	
C1	0.1858 (3)	0.5833 (3)	0.14354 (7)	0.0372 (5)	
C2	0.3828 (4)	0.5357 (3)	0.16995 (9)	0.0527 (6)	
H2A	0.4953	0.5136	0.1490	0.063*	
H2B	0.3611	0.4222	0.1872	0.063*	
C3	0.2221 (4)	0.7625 (3)	0.11730 (8)	0.0479 (6)	
H3A	0.0982	0.7945	0.1007	0.057*	
H3B	0.3326	0.7429	0.0957	0.057*	
C4	0.0121 (3)	0.6168 (3)	0.17747 (8)	0.0456 (6)	
H4A	−0.0107	0.5039	0.1950	0.055*	
H4B	−0.1146	0.6458	0.1615	0.055*	
C5	0.0674 (4)	0.7804 (3)	0.20945 (7)	0.0474 (6)	
H5	−0.0453	0.8011	0.2309	0.057*	
C6	0.2638 (4)	0.7272 (4)	0.23476 (9)	0.0570 (7)	
H6A	0.2993	0.8259	0.2558	0.068*	
H6B	0.2402	0.6133	0.2518	0.068*	
C7	0.4407 (4)	0.6974 (3)	0.20206 (8)	0.0514 (6)	
H7	0.5671	0.6664	0.2186	0.062*	
C8	0.4736 (4)	0.8731 (3)	0.17412 (9)	0.0539 (6)	
H8A	0.5135	0.9755	0.1938	0.065*	
H8B	0.5848	0.8522	0.1528	0.065*	
C9	0.2796 (4)	0.9257 (3)	0.14885 (8)	0.0471 (6)	
H9	0.3031	1.0398	0.1312	0.057*	
C10	0.1026 (4)	0.9556 (3)	0.18180 (9)	0.0513 (6)	
H10A	0.1348	1.0595	0.2016	0.062*	
H10B	−0.0221	0.9865	0.1653	0.062*	
C11	−0.0168 (4)	0.3554 (3)	0.10652 (8)	0.0476 (6)	
H11	−0.1226	0.3914	0.1258	0.057*	
C12	−0.0603 (4)	0.2091 (3)	0.07252 (8)	0.0490 (6)	
C13	0.0838 (4)	0.1560 (3)	0.04071 (8)	0.0554 (7)	
H13	0.2133	0.2121	0.0404	0.067*	
C14	0.0355 (6)	0.0188 (3)	0.00920 (9)	0.0681 (8)	
H14	0.1322	−0.0163	−0.0124	0.082*	
C15	−0.1551 (6)	−0.0648 (4)	0.00991 (9)	0.0731 (9)	
H15	−0.1870	−0.1577	−0.0109	0.088*	
C16	−0.2978 (5)	−0.0116 (4)	0.04126 (10)	0.0740 (9)	
H16	−0.4274	−0.0676	0.0416	0.089*	
C17	−0.2507 (4)	0.1241 (4)	0.07226 (9)	0.0610 (7)	
H17	−0.3490	0.1592	0.0935	0.073*	
N1	0.1537 (3)	0.4325 (2)	0.11053 (6)	0.0480 (5)	

### Atomic displacement parameters (Å^2^)

**Table:**

	*U*^11^	*U*^22^	*U*^33^	*U*^12^	*U*^13^	*U*^23^
C1	0.0310 (11)	0.0377 (11)	0.0429 (11)	−0.0010 (9)	0.0002 (9)	−0.0021 (9)
C2	0.0429 (13)	0.0489 (14)	0.0663 (16)	0.0083 (10)	−0.0030 (12)	0.0003 (12)
C3	0.0482 (14)	0.0500 (13)	0.0455 (12)	−0.0002 (11)	0.0021 (10)	0.0020 (11)
C4	0.0390 (12)	0.0479 (12)	0.0498 (13)	−0.0016 (11)	0.0036 (10)	0.0002 (10)
C5	0.0378 (12)	0.0591 (13)	0.0453 (12)	−0.0008 (11)	0.0085 (10)	−0.0021 (11)
C6	0.0570 (16)	0.0657 (16)	0.0482 (13)	−0.0072 (14)	−0.0023 (12)	0.0035 (12)
C7	0.0349 (12)	0.0594 (14)	0.0597 (14)	0.0034 (10)	−0.0114 (12)	0.0059 (12)
C8	0.0377 (13)	0.0601 (14)	0.0637 (15)	−0.0106 (12)	0.0016 (12)	0.0006 (13)
C9	0.0449 (13)	0.0422 (12)	0.0544 (13)	−0.0013 (10)	0.0013 (11)	0.0072 (10)
C10	0.0425 (14)	0.0469 (13)	0.0644 (14)	0.0046 (10)	−0.0021 (11)	−0.0056 (12)
C11	0.0428 (13)	0.0448 (12)	0.0552 (14)	0.0031 (11)	0.0020 (11)	−0.0013 (11)
C12	0.0612 (15)	0.0374 (11)	0.0486 (12)	−0.0024 (11)	−0.0054 (12)	0.0021 (10)
C13	0.0636 (16)	0.0423 (13)	0.0604 (15)	−0.0039 (11)	0.0016 (13)	0.0039 (12)
C14	0.103 (2)	0.0458 (14)	0.0551 (15)	0.0063 (16)	0.0102 (16)	−0.0020 (12)
C15	0.114 (3)	0.0505 (15)	0.0543 (15)	−0.0180 (17)	−0.0164 (18)	−0.0031 (13)
C16	0.083 (2)	0.0658 (17)	0.0730 (19)	−0.0313 (16)	−0.0089 (17)	−0.0048 (15)
C17	0.0646 (17)	0.0592 (15)	0.0592 (15)	−0.0128 (13)	−0.0007 (14)	−0.0028 (13)
N1	0.0479 (11)	0.0420 (10)	0.0541 (11)	−0.0021 (9)	0.0033 (10)	−0.0048 (9)

### Geometric parameters (Å, º)

**Table:**

C1—N1	1.471 (3)	C8—C9	1.511 (3)
C1—C3	1.516 (3)	C8—H8A	0.9700
C1—C4	1.529 (3)	C8—H8B	0.9700
C1—C2	1.536 (3)	C9—C10	1.522 (3)
C2—C7	1.543 (3)	C9—H9	0.9800
C2—H2A	0.9700	C10—H10A	0.9700
C2—H2B	0.9700	C10—H10B	0.9700
C3—C9	1.541 (3)	C11—N1	1.240 (3)
C3—H3A	0.9700	C11—C12	1.480 (3)
C3—H3B	0.9700	C11—H11	0.9300
C4—C5	1.547 (3)	C12—C17	1.375 (3)
C4—H4A	0.9700	C12—C13	1.381 (3)
C4—H4B	0.9700	C13—C14	1.390 (3)
C5—C10	1.514 (3)	C13—H13	0.9300
C5—C6	1.525 (3)	C14—C15	1.371 (5)
C5—H5	0.9800	C14—H14	0.9300
C6—C7	1.517 (3)	C15—C16	1.366 (4)
C6—H6A	0.9700	C15—H15	0.9300
C6—H6B	0.9700	C16—C17	1.371 (4)
C7—C8	1.519 (3)	C16—H16	0.9300
C7—H7	0.9800	C17—H17	0.9300
			
N1—C1—C3	107.34 (16)	C2—C7—H7	110.1
N1—C1—C4	116.69 (17)	C9—C8—C7	111.07 (19)
C3—C1—C4	108.70 (17)	C9—C8—H8A	109.4
N1—C1—C2	107.17 (17)	C7—C8—H8A	109.4
C3—C1—C2	108.63 (18)	C9—C8—H8B	109.4
C4—C1—C2	108.08 (18)	C7—C8—H8B	109.4
C1—C2—C7	110.58 (18)	H8A—C8—H8B	108.0
C1—C2—H2A	109.5	C8—C9—C10	110.04 (19)
C7—C2—H2A	109.5	C8—C9—C3	108.37 (19)
C1—C2—H2B	109.5	C10—C9—C3	108.3 (2)
C7—C2—H2B	109.5	C8—C9—H9	110.0
H2A—C2—H2B	108.1	C10—C9—H9	110.0
C1—C3—C9	111.33 (17)	C3—C9—H9	110.0
C1—C3—H3A	109.4	C5—C10—C9	110.23 (19)
C9—C3—H3A	109.4	C5—C10—H10A	109.6
C1—C3—H3B	109.4	C9—C10—H10A	109.6
C9—C3—H3B	109.4	C5—C10—H10B	109.6
H3A—C3—H3B	108.0	C9—C10—H10B	109.6
C1—C4—C5	110.57 (18)	H10A—C10—H10B	108.1
C1—C4—H4A	109.5	N1—C11—C12	123.3 (2)
C5—C4—H4A	109.5	N1—C11—H11	118.4
C1—C4—H4B	109.5	C12—C11—H11	118.4
C5—C4—H4B	109.5	C17—C12—C13	118.8 (2)
H4A—C4—H4B	108.1	C17—C12—C11	119.1 (2)
C10—C5—C6	110.3 (2)	C13—C12—C11	122.1 (2)
C10—C5—C4	109.06 (18)	C12—C13—C14	120.1 (3)
C6—C5—C4	107.88 (19)	C12—C13—H13	120.0
C10—C5—H5	109.9	C14—C13—H13	120.0
C6—C5—H5	109.9	C15—C14—C13	120.0 (3)
C4—C5—H5	109.9	C15—C14—H14	120.0
C7—C6—C5	110.52 (19)	C13—C14—H14	120.0
C7—C6—H6A	109.5	C16—C15—C14	119.9 (3)
C5—C6—H6A	109.5	C16—C15—H15	120.0
C7—C6—H6B	109.5	C14—C15—H15	120.0
C5—C6—H6B	109.5	C15—C16—C17	120.2 (3)
H6A—C6—H6B	108.1	C15—C16—H16	119.9
C6—C7—C8	109.8 (2)	C17—C16—H16	119.9
C6—C7—C2	108.4 (2)	C16—C17—C12	121.1 (3)
C8—C7—C2	108.38 (19)	C16—C17—H17	119.5
C6—C7—H7	110.1	C12—C17—H17	119.5
C8—C7—H7	110.1	C11—N1—C1	120.97 (19)

## References

[bb1] Bruker (2004). *SMART* and *SAINT* Bruker AXS Inc., Madison, Wisconsin, USA.

[bb3] Jin, X.-D., Jin, Y.-H., Zou, Z.-Y., Cui, Z.-G., Wang, H.-B., Kang, P.-L., Ge, C.-H. & Li, K. (2011). *J. Coord. Chem.* **64**, 1533–1543.

[bb4] Sheldrick, G. M. (1996). *SADABS* University of Göttingen, Germany.

[bb5] Sheldrick, G. M. (2008). *Acta Cryst.* A**64**, 112–122. 10.1107/S0108767307043930 18156677

[bb6] Zhao, G.-L. & Feng, Y.-L. (2005). *Z. Kristallogr. New Cryst. Struct.* **220**, 197–198.

